# RNA-protein correlation of liver toxicity markers in HepaRG cells

**DOI:** 10.17179/excli2019-2005

**Published:** 2020-01-17

**Authors:** Albert Braeuning, Almut Mentz, Felix F. Schmidt, Stefan P. Albaum, Hannes Planatscher, Jörn Kalinowski, Thomas O. Joos, Oliver Poetz, Dajana Lichtenstein

**Affiliations:** 1 German Federal Institute for Risk Assessment, Dept. Food Safety, Berlin, Germany; 2 Center for Biotechnology (CeBiTec), Universität Bielefeld, Bielefeld, Germany; 3 Signatope GmbH, Reutlingen, Germany; 4 NMI Natural and Medical Sciences Institute at the University of Tübingen, Tübingen,Germany

**Keywords:** liver toxicity, in vitro testing, hepatocytes, relative potency factors, omics

## Abstract

The liver is a main target organ for the toxicity of many different compounds. While in general, *in vivo* testing is still routinely used for assessing the hepatotoxic potential of test chemicals, the use of *in vitro* models offers advantages with regard to throughput, consumption of resources, and animal welfare aspects. Using the human hepatoma cell line HepaRG, we performed a comparative evaluation of a panel of hepatotoxicity marker mRNAs and proteins after exposure of the cells to 30 different pesticidal active compounds comprising herbizides, fungicides, insecticides, and others. The panel of hepatotoxicity markers included nuclear receptor target genes, key players of fatty acid and bile acid metabolism-related pathways, as well as recently identified biomarkers of drug-induced liver injury. Moreover, marker genes and proteins were identified, for example, S100P, ANXA10, CYP1A1, and CYP7A1. These markers respond with high sensitivity to stimulation with chemically diverse test compounds already at non-cytotoxic concentrations. The potency of the test compounds, determined as an overall parameter of their ability to deregulate marker expression *in vitro*, was very similar between the mRNA and protein levels. Thus, this study does not only characterize the response of human liver cells to 30 different pesticides but also demonstrates that hepatotoxicity testing in human HepaRG cells yields well comparable results at the mRNA and protein levels. Furthermore, robust hepatotoxicity marker genes and proteins were identified in HepaRG cells.

## Abbreviations

ACN, acetonitrile; AHR, aryl hydrocarbon receptor; AOP, adverse outcome pathway; CAR, constitutive androstane receptor; CYP, cytochrome P450; DMSO, dimethyl sulfoxide; FBS, fetal bovine serum; FA, formic acid; LC-MS, liquid chromatography-mass spectrometry; LOAEL, lowest observed adverse effect level; NOAEL, no observed adverse effect level; PBS, phosphate-buffered saline; PXR, pregnane-X-receptor; RPF, relative potency factor; TXP, Triple X Proteomics; WST, water-soluble tetrazolium

## Introduction

The liver is a main target organ of a plethora of toxicants, including for example, certain drugs, industrial chemicals, pesticidal active compounds, natural toxins, environmental contaminants (Adams et al., 2005[1]; EFSA Scientific Committee et al., 2019[17]; Alexander, 2012[3]). In classic toxicological testing, hepatotoxicity of a test compound is determined in *in vivo* studies mainly conducted in rodents, based on detailed histopathological examination of tissue specimens after prolonged, repeated exposure to the test chemical (OECD, 2019[52]; Pradeep et al., 2016[60]). Hepatic responses to exposure to xenobiotics can be manifold. Often, adaptive responses are observed, as exemplified by hepatocyte hypertrophy and enlargement of the smooth endoplasmatic reticulum, which is frequently detected as a consequence of the induction of hepatocellular drug-metabolizing capacities following activation of drug metabolism-regulating nuclear receptors by foreign compounds (Maronpot et al., 2010[46]; Schulte-Hermann, 1979[63]). Such responses include, for example, regulation of gene transcription by the constitutive androstane receptor (CAR), the pregnane-X-receptor (PXR), or the aryl hydrocarbon receptor (AHR) (Maronpot et al., 2010[46]). The most prominent target genes of these receptors come from the cytochrome P450 (CYP) superfamily of genes encoding important phase I drug-metabolizing enzymes (Tompkins and Wallace, 2007[72]; Waxman, 1999[78]). Reactive compounds or CYP metabolism-generated intermediates, such as radicals and electrophiles, can cause oxidative stress to hepatocytes followed by cell death, whereas more subtle manifestations of toxicity often comprise alterations in crucial metabolic pathways of the hepatocytes. For example, disturbance of the balance of fatty acid synthesis and degradation may result in fatty liver cells, potentially giving rise to progression of hepatic steatosis to liver inflammation, cirrhosis, and cancer (Basaranoglu et al., 2013[9]; Leung and Nieto, 2013[43]; Sturgill and Lambert, 1997[70]). Another example is the disruption of bile acid synthesis and excretion leading to cholestatic livers (Padda et al., 2011[54]; Waxman, 1992[79]). Key genes and proteins affected by toxicants in such pathways have, in some cases, been assembled to so-called adverse outcome pathways (AOPs) which describe causal relationships of molecular events leading to adverse responses at the organ level (Ankley et al., 2010[4]; Leist et al., 2017[42]; Vinken, 2013[75]).

Animal studies are ethically disputed, rather cost- and time-consuming, especially in the case of repeated-dose studies, and questioned for their relevance to humans, due to possible species differences (Graham and Lake, 2008[21]; Hackam and Redelmeier, 2006[25]; Martignoni et al., 2006[47]). Thus, there is a need for establishing *in vitro* approaches using human cells in order to circumvent the aforementioned drawbacks. This holds especially true with respect to the testing of the effects of chemical mixtures. Here, testing of the multitude of possible combinations of individual compounds is not feasible using animal-based approaches. A plethora of *in vitro* hepatotoxicity studies have been conducted using either primary hepatocytes or permanent hepatoma-derived cell lines. Measured endpoints range from simple cell viability assays to the measurement of complex metabolic endpoints, transcriptional responses or proteomic alterations (Bale et al., 2014[7]; Kyffin et al., 2018[39]; Soldatow et al., 2013[68]). 

Especially transcriptomic signatures have been used to help characterizing the toxicological mode of action of chemicals and to classify test compounds according to their mechanisms of toxicity. For example, a lot of research has been performed to distinguish genotoxic from non-genotoxic carcinogens using transcript-based omics approaches (Ellinger-Ziegelbauer et al., 2005[18]; Jennen et al., 2010[31]; Lee et al., 2013[40]). In addition, panels of common marker genes for hepatotoxicity have been identified from omics data using bioinformatic methods (Albrecht et al., 2019[2]; Grinberg et al., 2018[22]). In contrast to the lot of work that has been performed at the mRNA level, proteomic data on hepatotoxicity have been studied less extensively.

Even though mRNAs are generally translated in proteins, a direct correlation of transcript and protein levels of a certain gene cannot be expected, because additional layers of cellular regulation such as alterations in translation efficiency or protein stability may considerably affect the outcome of protein level determination (Gry et al., 2009[23]). Knowledge of the correlation of the RNA and protein level alterations can help to improve our understanding of *in vitro* systems for hepatocellular toxicity, and contribute to assess the relevance of RNA-based data sets. Therefore, we here performed a comparative characterization of transcript- and protein-level responses using 30 different pesticidal active compounds as test items. The human hepatocarcinoma cell line HepaRG was chosen as a test system, based on the high degree of similarity of these cells with human hepatocytes (Kanebratt and Andersson, 2008[32]).

## Materials and Methods

### Chemicals

Cyproconazole, epoxiconazole, and prochloraz were obtained from BASF or Syngenta, respectively. The batches used identical to what has been used in a previous study by Seeger et al. (2019[66]). All other pesticidal active compounds were purchased from Sigma (Taufkirchen, Germany) in the highest available purity, dissolved in DMSO (purity > 99 %; Carl Roth, Karlsruhe, Germany), and stored at -20 °C until further use. *In vitro* testing was performed up to the highest possible concentrations, limited by the compound-specific solubility in cell culture medium. All other chemicals were obtained from Sigma or Merck (Darmstadt, Germany) in the highest available purity.

### Cell culture

Human HepaRG hepatocarcinoma cells were purchased from Biopredic International (Saint Grégoire, France) and seeded according to the manufacturer's protocol at densities of 9,000, 100,000, or 200,000 cells/well in 96-well, 12-well, or 6-well plates, respectively. For cultivation William's Medium E with 2 mM glutamine (PAN-Biotech, Aidenbach, Germany), 10 % (v/v) fetal bovine serum (FBS; FBS Good Forte EU approved; PAN-Biotech), 100 U/ml penicillin and 100 µg/ml streptomycin (Capricorn Scientific, Ebsdorfergrund, Germany), 0.05 % human insulin (PAA Laboratories GmbH, Pasching, Austria) and 50 µM hydrocortisone hemisuccinate (Sigma) were used. Cells were cultured under standard cell culture conditions (37 °C in a humidified atmosphere with 5 % CO_2_). Cells were differentiated for 14 days, followed by 14 days in medium additionally containing 1.7 % DMSO. Differentiated HepaRG cells were pre-adapted to treatment medium (culture medium containing only 2 % FBS and 0.5 % DMSO) for 48 h prior to exposure in treatment medium for 24 h, with a final DMSO concentration of 0.5 %. Cell identity and differentiation status were checked microscopically. Differentiated HepaRG cells exhibit characteristic, unique morphological features, as provided by the manufacturer´s specifications as well as described in several publications (Cerec et al., 2007[11]; Guillouzo et al., 2007[24]; Parent et al., 2004[56]). Additionally, cells were routinely checked for the absence of mycoplasma contaminations.

### Cell viability analysis

Viability of cells treated with pesticidal active compounds was analyzed in HepaRG cells using the WST-1 cell assay (Sigma-Aldrich, St. Louis, USA) as described by Luckert et al. (2018[45]). In brief, cells were seeded in 96-well plates and incubated with the respective test compound. Triton X-100 (0.01 %) served as a positive control for cytotoxicity. One hour before the end of incubation, 10 µl WST-1 reagent was added to each well containing 100 µl medium and incubated again for one hour. Afterwards, absorbance was measured at 450 nm and corrected by values measured at the reference wavelength of 620 nm, using an Infinite M200 Pro plate reader (Tecan, Männedorf, Switzerland). At least three independent biological replicates, each with six technical replicates per condition, were run.

### Analysis of mRNA expression levels

Cells were seeded in 12-well plates and incubated for 24 h with the respective test compounds or solvent control (0.5 % DMSO). Afterwards, cells were washed twice with ice-cold phosphate-buffered saline (PBS) and lysed using 350 µl RLT buffer per well (RNeasy Mini Kit; Qiagen, Hilden, Germany) containing 3.5 µl β-mercaptoethanol. RNA was isolated according to the manufacturer's protocol. Additionally, potential DNA contaminations were removed by DNase digestion (RNase-Free DNA Set; Qiagen). For higher yields, RNA was eluted twice in 30 µl H_2_O. RNA was quantified at 260 nm using a Tecan spectrometer (Tecan, Männedorf, Switzerland). For purity evaluation the 260 nm/280 nm absorbance ratio was determined and ratios > 1.8 were considered an acceptable RNA purity. RNA integrity was checked by micro-gel electrophoresis using the Agilent RNA 6000 Nano Kit in a Bioanalyzer (Agilent Technologies, Santa Clara, CA, USA). Only RNAs with calculated RIN (RNA Integrity Number) values > 9 were used (quality ranging from 10, i.e. highly intact RNA to 1, i.e. completely degraded RNA). RNA samples were stored at -80 °C. Primer design for quantitative RT-PCR experiments (Supplementary Table 1) was performed by using qPrimerDepot (Cui et al., 2006[12]) and RTPrimerDB (Lefever et al., 2008[41]) and was validated by melting curve analysis. Relative expression from treatment procedures against DMSO-treated Reference RNA of all 47 genes was analyzed using 30 ng total RNA and the SensiFAST™ SYBR® No-ROX Kit (Bioline, Luckenwalde, Deutschland). All measurements were conducted in triplicates from biological replicates on a LightCycler® 96 System (Roche, Basel, Schweiz) applying sample maximization as experimental plate design. Efficiency of gene amplification was calculated for each run with LinRegPCR (Ramakers et al., 2003[61]; Ruijter et al., 2009[62]). For calculation of relative expression - including normalization by three reference genes - the software REST384 (Pfaffl, 2001[57]; Pfaffl et al., 2002[58]) was applied.

### Analysis of protein expression levels

Cells were seeded in 12-well plates and incubated for 24 h with the respective test compounds or solvent control (0.5 % DMSO). Afterwards, cells were washed twice with ice-cold PBS and lysed using 400 µl/well lysis buffer containing 1 % NP-40, 0.01 % sodium dodecyl sulfate (SDS); 0.15 M sodium chloride; 0.01 M sodium phosphate; 2 mM ethylenediaminetetraacetic acid (EDTA); and 2.5 U/ml benzonase at pH 7.2. After 1 h of shaking at 4 °C, lysates were collected and stored at -80 °C. After lysis, the protein concentration was determined using the bicinchoninic acid assay (Thermo Fisher Scientific, Waltham, USA). The manufacturer's protocol was used for this purpose. The samples were diluted 1:5 before analysis and the concentrations determined with the microplate reader BioTek ELx808 (BioTek, Winooski, USA) at a wavelength of 562 nm. This was followed by tryptic proteolysis. Triethanolamine (TEA) with a final concentration of 50 mM and lysis buffer was added to the samples, which were then denatured at 99 °C for five minutes. After cooling to room temperature, tris(2-carboxyethyl) phosphine (TCEP; final concentration 5 mM) was added. After shaking for 30 sec, iodoacetamide (IAA) with a final concentration of 10 mM was added and samples were shaken again for 30 min at room temperature in the dark. Proteolysis was initiated with the addition of trypsin (trypsin:protein ratio 1:40) and the samples were digested at 37 °C for either 2 or 16 h. To stop digestion, phenylmethanesulfonyl fluoride (PMSF) with a final concentration of 1 mM was added. The samples were centrifuged for 10 min at 13,000 x *g* to remove cell debris. Before the samples were analyzed by liquid chromatography-mass spectrometry (LC-MS), immunoprecipitation was performed. The sample was mixed with PBS containing CHAPS (3-[(3-cholamidopropyl) dimethylammonio] -1-propanesulfonate), internal isotopically labeled standard peptide and the respective antibodies to enrich the target analytes. Special antibodies (called Triple X Proteomics; TXP) were used. These TXP antibodies specifically recognize the last four amino acids of the C-terminus. Thus, an antibody can enrich not only one peptide but whole peptide groups at once (Poetz et al., 2009[59]). The complete immunoprecipitation was performed as described in Weiß et al. (2018[80]).

An Acclaim PepMap RSLC C18 (75 μm I.D. x 150 mm, 2 μm, Thermo Fisher Scientific, Waltham, USA) analytical column and a trapping column Acclaim PepMap 100 C18 μ precolumn (0.3 mm I.D. x 5 mm, 5 μm, Thermo Fisher Scientific, Waltham, USA) were used for LC-MS analysis. The samples were measured in parallel reaction monitoring (PRM). The method duration was either 20 min (for the CYP 17-plex with a flow rate of 0.3 µl/min and an oven temperature of 40 °C) or 10 min (for all other multiplex assays with a flow rate of 1 µl/min and an oven temperature of 55 °C). Eluent A (aqueous phase) consisted of LC-MS grade water, with 0.1 % formic acid (FA) added. Eluent B (organic phase) of 80 % acetonitrile (ACN) and 20 % LC-MS grade water with 0.1 % FA. As loading buffer, 2 % ACN with LC-MS grade water and 0.05 % trifluoroacetic acid (TFA) was used. The evaluation of the data was carried out via Skyline 4.2.0. 19072. The ratio of the obtained endogenous signal and the internal isotopically labeled standard peptide was calculated. For each analyte, only the most intense fragment ion (quantifier ion) was used. For each test substance, cells were treated three times. Each sample was measured and mean values were calculated from the biological replicates. Fold changes were obtained by referencing the results of the treatments to the solvent control.

### Determination of compound potency

As recommended by EFSA (2011[14]; EFSA Scientific Committee et al., 2017[16]; Kortenkamp et al., 2009[38]) and described previously (Kienhuis et al., 2015[33]; Staal et al., 2018[69]), a benchmark dose (BMD) approach was used which is currently considered the most appropriate approach for deriving reference points. One kind of reference point obtained by this method is the relative potency of a compound. By scaling the concentration-response of one compound relatively against the concentration-response of another, i.e. the reference compound, the relative potency factor (RPF) was obtained as a scaling factor. Usually, RPFs are computed for a single parameter of interest, e.g. cytotoxicity or the induction of expression of a specific target gene. In the context of omics data, this would lead to a plethora of different RPFs and constitute a time-consuming and inefficient approach. To overcome this drawback we developed a strategy which considers all analytes obtained during transcriptomic or proteomics analysis applying confidence interval statistics. For this purpose, the width of the confidence interval of transcriptomic alterations as an equivalent for the overall degree of transcriptomic alterations by one compound was used. Thus, half the width of confidence interval - mathematically defined as the absolute error e and measure of the accuracy of the estimation of a parameter - is defined as the potency factor (equation 1).


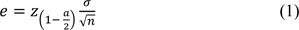


There, e denotes the absolute error or potency factor, z denotes the (1-α/2)-quantile of standard normal distribution and α = 0.05, i.e. including 95 % of the values. The parameter σ reflects the standard deviation and n is the number of data points per experiment. Due to different tested concentrations a correction for compound concentration by which effect was achieved is additionally required. Thus, e was furthermore divided into the administered concentration per compound. The relative potencies were computed for all compounds against each other. The software tool Genesis 1.8.1 (Sturn et al., 2002[71]) was applied for Cluster analysis.

## Results

### Selection of hepatotoxicity markers and test compounds

A panel of candidate hepatotoxicity markers was assembled based on data from the literature and own previous studies (Grinberg et al., 2018[22]; Seeger et al., 2019[66]). The panel included various nuclear receptor target genes (e.g. from the cytochrome P450 (CYP) superfamily), key players of fatty acid and bile acid metabolism-related pathways as compiled in the AOPs for steatosis and cholestasis (e.g. (Mellor et al., 2016[49]; Vinken, 2015[74]; Vinken et al., 2013[76]), as well as genes recently identified as biomarkers for drug-induced liver injury (e.g. Albrecht et al., 2019[2]; Grinberg et al., 2018[22]). Following an initial screening approach with several hepatotoxic pesticides, some functionally redundant entries with very similar regulation (e.g. closely related CYP genes known to be affected by identical transcription factors), as well as some genes not expressed in HepaRG cells or not influenced by any of the test compounds were removed from the panel, resulting in a final number of 51 hepatotoxicity markers used for subsequent analyses. The selected markers are listed in Table 1(Tab. 1).

Pesticidal active compounds were selected as test chemicals with well-known toxicological profiles. In order to cover a broad spectrum of exposure-relevant pesticides, 30 different substances were chosen for *in vitro* testing (Table 2(Tab. 2)). These belong to various chemical classes of pesticides, namely: anilinopyrimidines, benzimidazoles, carboxamides / dicarboximides, dithiocarbamates, imidazoles, morpholines, neonicotinoids, organophosphates, phthalimides, phenylpyrazoles / pyrazoles, strobilurins, triazoles, and quaternary ammonium compounds (Table 2(Tab. 2)), with liver as their main target organ *in vivo*.

### Cell viability testing

Using the WST-1 assay, all compounds were screened in HepaRG cells for 24 h regarding their cytotoxic potential, for the evaluation of suitable test concentrations for transcriptomic and proteomic analysis (data not shown). The highest non-cytotoxic concentrations from the WST-1 assay were selected for subsequent transcriptomic and proteomic analysis and are listed in Table 2(Tab. 2).

### Transcriptomic and proteomic analysis of active compounds

**Figure 1:**
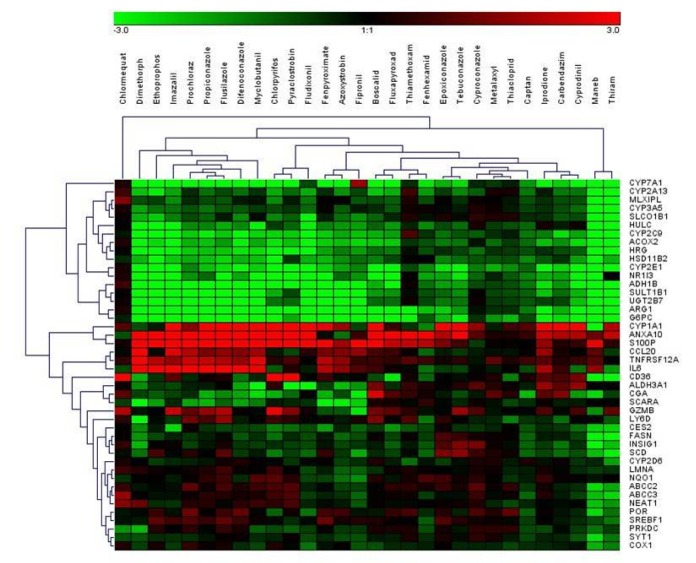
Hierarchical clustering (average linkage) method shows groups of downregulated genes (green) and upregulated genes (red) over all substances. Chlormequat, maneb, and thiram cluster most distant from all other compounds.

**Figure 2:**
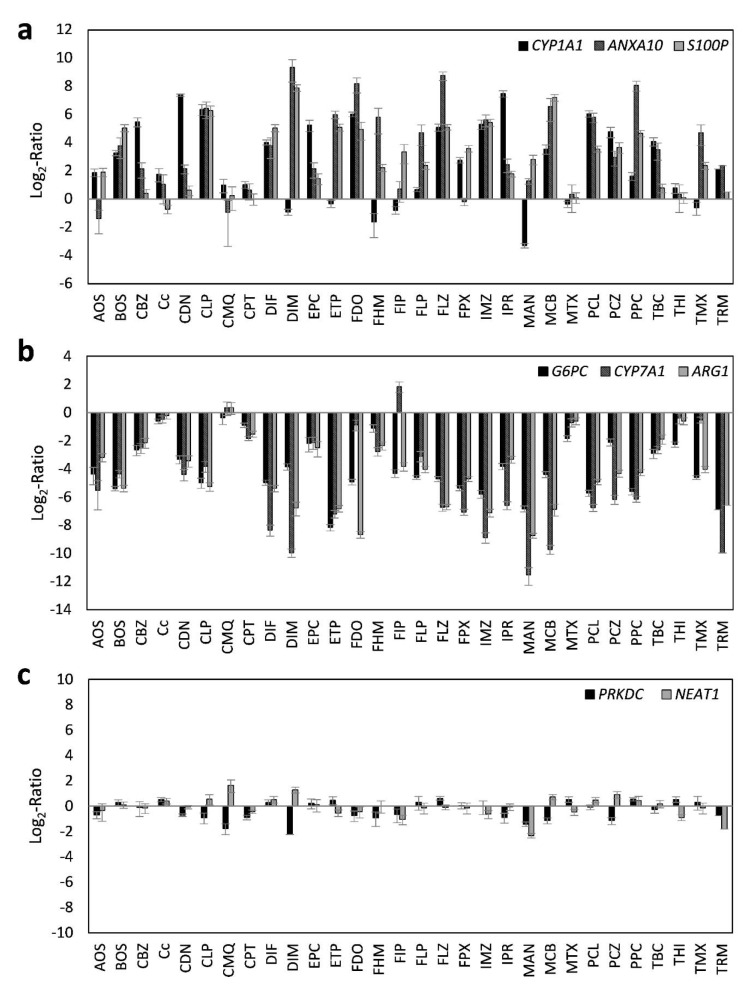
Representative deregulated transcripts in HepaRG cells after 24 h of treatment with 30 different pesticidal active compounds. Preferentially upregulated transcripts (a), preferentially downregulated transcripts (b), and transcripts with only weak alterations (c) are shown.

Using the selected highest non-toxic concentration of each compound, mRNA expression of the 44 hepatotoxicity marker genes was determined in HepaRG cells. The complete dataset is contained in Supplementary Table 2. In total, a heterogeneous response was recorded at the mRNA level, involving up- and downregulation as well as pronounced and more subtle responses. Data are summarized in Figure 1(Fig. 1). Individual examples of regulation of selected genes are provided in Figure 2(Fig. 2), showing the results for the preferentially upregulated genes e.g. *S100P*, the preferentially downregulated genes *CYP7A1*, and for e.g. *NEAT1* showing only weak alterations upon pesticide treatment. 

**Figure 3:**
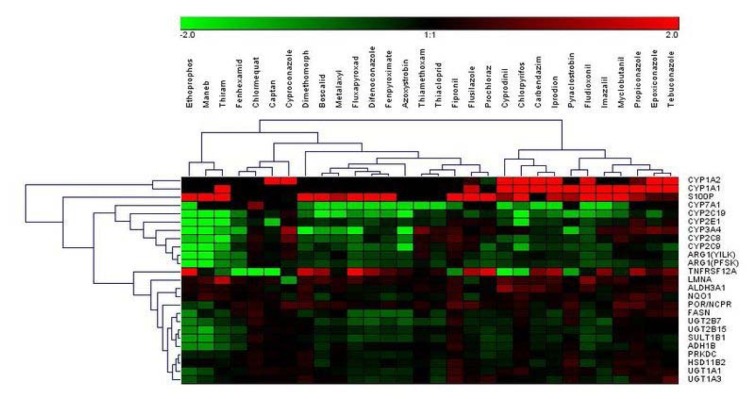
Hierarchical clustering method (average linkage) shows groups of downregulated proteins (green) and upregulated proteins (red) over all substances. Ethoprophos, maneb, and thiram cluster most distant from all other compounds.

**Figure 4:**
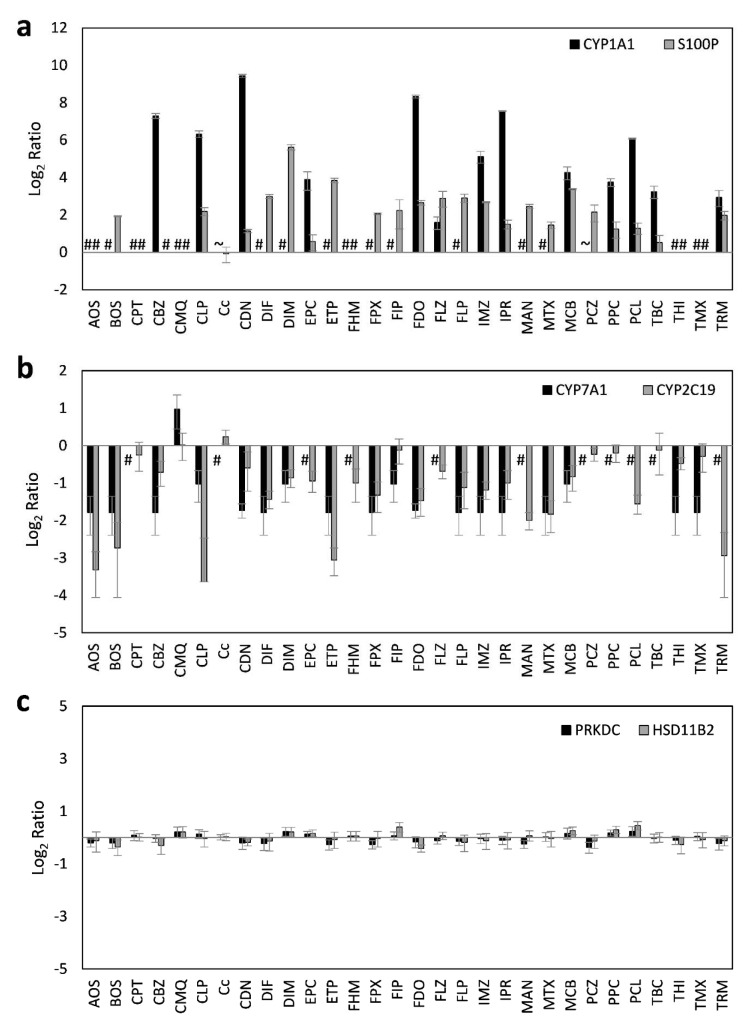
Representative deregulated proteins in HepaRG cells after 24 h of treatment with 30 different pesticidal active compounds. Preferentially upregulated proteins (a), preferentially downregulated proteins (b), and proteins with only weak alterations (c) are shown. #, no endogenous protein levels detectable. Data below the lower limit of quantification (LLOQ) of 0.075 fmol/µg Protein (CYP1A1) are marked with ~.

In order to obtain correlating data at the protein level, a set of multiplexed targeted MS-based assays was used, consisting of existing and newly developed assays. In total, quantitative determination of 24 proteins was performed, with 17 proteins corresponding to important marker genes already assayed at the mRNA level (Table 1(Tab. 1)). HepaRG cells were incubated with the same concentrations of pesticidal active compounds as used for the mRNA analyses. Total results can be found in Supplementary Table 3 and are visualized in Figure 3(Fig. 3). Selected protein level data are depicted in Figure 4(Fig. 4).

### RNA-protein correlation of hepatotoxicity markers in HepaRG cells

**Figure 5:**
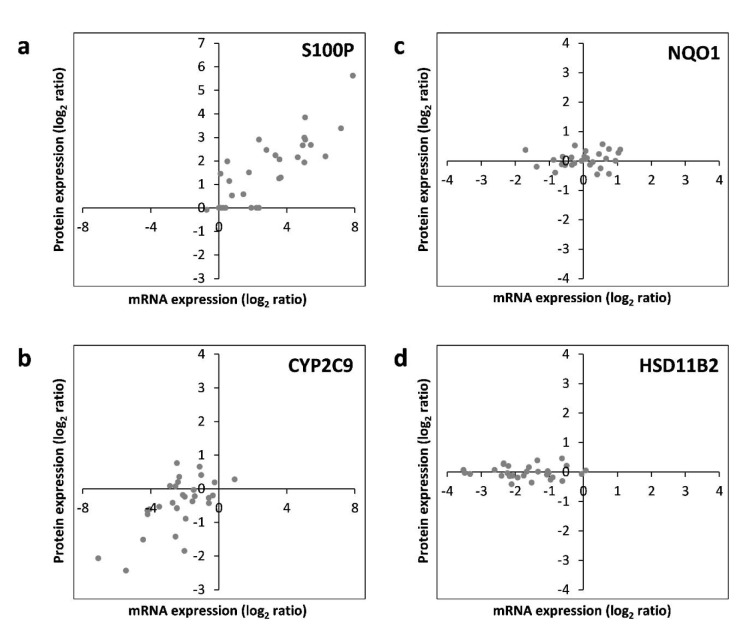
RNA-protein correlation of different hepatotoxicity markers assayed in HepaRG cells treated with 30 pesticidal active compounds for 24 h. Representative examples of common upregulation (S100P; panel a), common downregulation (CYP2C9; panel b), common lack of regulation (NQO1; panel c), as well as regulation at the mRNA but not protein level (HSD11B2; panel d) are depicted. Each dot represents the mean data from mRNA and protein quantification resulting from treatment of HepaRG cells with an individual chemical.

Subsequently, correlation of the data obtained individually at the mRNA and protein levels was assessed. For each of the 17 hepatotoxicity markers for which mRNA and protein data were available, correlations were analyzed. This is exemplarily depicted for S100P, NQO1, CYP2C9, and HSD11B2 in Figure 5(Fig. 5). The full datasets are available in Supplementary Tables 2 and 3. Similar to S100P, CYP1A1, and TNFRSF12A also predominantly showed a consistent upregulation at the mRNA and protein levels by most chemicals (Figure 5(Fig. 5); and data not shown). Predominant downregulation at both, the mRNA and protein levels was observed for ADH1B, ARG1, CYP2C9, CYP2E1, SULT1B1, and UGT2B7 (Figure 5(Fig. 5); and data not shown). A tendency for that behavior was also seen for CYP7A1; however, the fact that protein levels of CYP7A1 were below the LOQ in several samples impeded comprehensive comparative analysis of this marker gene/protein (data not shown). A positive correlation between mRNA and protein data was also visible for ALDH3A1, FASN, and POR, even though a considerable number of deviations (8, 9, and 12 out of 30 compounds, respectively) from consistent up- or downregulation in the two datasets were recorded (data not shown). A comparable weak regulation of NQO1, without clear preference for up- or downregulation by a majority of compounds, was seen at the mRNA as well as protein levels (Figure 5(Fig. 5)). Inverse correlation of the direction of regulation was only observed for 2 of the 17 mRNAs/proteins, namely LMNA and PRKDC (data not shown). For HSD11B2, downregulation of mRNA expression but no clear-cut regulation at the protein was visible, possibly indicating a delayed response at the protein level (Figure 5(Fig. 5)). Overall, these findings point towards an overall good accordance of the responses obtained at the transcriptional and proteomic levels.

### Omics-derived relative potency factors

**Figure 6:**
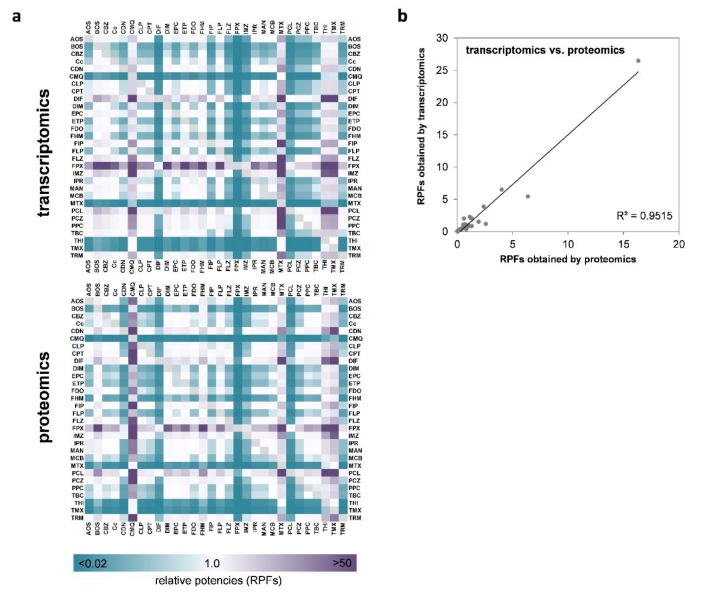
Comparison of relative potencies obtained via transcriptomics and proteomics (a). The scale bar denotes higher or lower RPFs of a compound, as compared to another compound, in purple and blue, respectively. (b) Analysis of correlation of transcriptomic and proteomic RPFs. Each dot represents the RPF derived at the mRNA and protein levels for treatment with an individual compound.

In addition to the qualitative correlation of the direction of responses at the level of individual genes or proteins, we were also interested in comparing the overall relative potencies of the test compounds to alter RNA and protein levels on a broader basis. For this purpose, we decided to use relative potency factors derived by a benchmark dose approach. Such potency factors constitute a useful compound-characterizing parameter that is usually related to one specific biological endpoint, e.g. *CYP3A4* expression. To adapt this approach to multi-endpoint data as resulting from omics analysis, the development of a strategy that considers the whole dataset needs to be developed. For this purpose, we developed a method that uses the width of the confidence interval of transcriptomic or proteomic alterations as an equivalent for the overall degree of transcriptomic alterations by one compound, independent of the nature of the specific genes or proteins that were subject to regulation. The mathematical background of the total omics RPF approach is described in detail in the 'Materials and Methods' section, paragraph 'Determination of compound potency'. This way, we were able to deduce RPFs for all compounds, based on their ability to deregulate gene or protein expression in HepaRG cells. Heatmap visualization of the RPFs is presented in Figure 6A(Fig. 6). For a full table of RPFs, please refer to Supplementary Table 4. When comparing the RPFs derived from transcriptomic and proteomic analysis, it became evident that a high degree of concordance was present in the two datasets, i.e. that compounds which strongly altered the transcriptomic pattern of HepaRG cells were also potent at the protein level. Correlation analysis resulted in an R^2^ value of 0.9515 (Figure 6B(Fig. 6)) with difenoconazole (DIF), fenpyroximate (FPX), and pyraclostrobin (PCL) being identified as the most potent compounds.

## Discussion

The present work on the regulation of hepatotoxicity markers in HepaRG cells shows an overall good correlation between changes at the mRNA and protein levels, suggesting a considerable degree of comparability of the data obtained at the mRNA and protein levels. The broad response of HepaRG cells to the various hepatotoxic compounds underlines their usefulness as an *in vitro* model system for human liver cells. Interestingly, many genes show a uniform-type response, which means that they are generally either down- or upregulated by most of the test compounds. This is remarkable, as there is high chemical diversity among the thirty test compounds, connected to a variety of different molecular targets resulting in different mechanisms of toxicity. For some genes, it might be expected to observe such a pattern. Such an example could be a nuclear receptor target gene with low constitutive expression and pronounced inducibility, for example the AHR target *CYP1A1* (Barouki et al., 2007[8]; Schulthess et al., 2015[64]). As not only classic AHR ligands such as dioxins, but also weaker, non-prototypical ligands can affect *CYP1A1* expression, an induction by many test compounds is plausible. Of note, weak activation of AHR-dependent transcription has been published for some of the compounds used here, for example propiconazole (Knebel et al., 2018[37], 2019[35]), tebuconazole (Knebel et al., 2019[36]), prochloraz (Heise et al., 2015[28], 2018[27]; Vinggaard et al., 2006[73]). 

Nonetheless, the hepatotoxicity marker panel presented here contains a substantial number of genes or proteins that are not directly linked to up-regulation by xenobiotic-activated nuclear receptors; and many of these genes show a tendency for regulation in a specific direction by mechanistically very different compounds. This indicates that the respective genes represent rather general and robust markers of hepatocellular stress, both at the mRNA and protein level. Responses were recorded at non-cytotoxic concentrations indicating a higher sensitivity of these transcript-based markers, as compared to classic cell vitality or cytotoxicity assays. It should be noted that transcriptional classifiers for hepatotoxicity have been subject of previous research projects, using different cellular models and/or test compounds (Grinberg et al., 2018[22]; Seeger et al., 2019[66]). Using the total degree of deregulation of such non-pathway-specific hepatocellular toxicity markers as a global parameter, it may be possible to estimate the hepatotoxic potential of test compounds *in vitro*. Therefore, we assessed whether the determined *in vitro* RPFs would correlate with the potencies of the compound to exert hepatotoxicity *in vivo*. Here, no pronounced correlation of the *in vitro *RPFs with *in vivo* LOAELs/NOAELs (no/lowest observed adverse effect levels), the toxicity parameters classically deduced from *in vivo* studies, was observable. However, that outcome may rather be expected for different reasons: first, the NOAELs and LOAELs bear a high level of uncertainty related to study design. This involves, amongst others, parameters like duration of exposure and the choice of dose levels and species. Benchmark approaches may help to improve the latter issue, but one has to bear in mind that such an approach is not compatible with the dosing schemes of many published studies. Of note, RPFs and NOAELs/LOAELs derived from the same studies do not necessarily show a high degree of correlation (EFSA Panel on Plant Protection Products and their Residues, 2009[15]). Second, species differences may exist regarding the sensitivity to certain hepatotoxicants between rodents and humans and therefore the predictivity of animal NOAELs/ LOAELs for humans may be limited. Of note, also the NOAELs/LOAELs of different non-human species show considerable variance. For example, dioxin toxicity is at great variance between species (Scientic Committee on Food, 2000[65]). Species differences are especially relevant for agonists of nuclear receptors, where often considerable differences between species are observed. For example, hepatic effects of exposure to cyproconazole in rodents are thought to be mediated by CAR, whereas humanization of the receptor in transgenic mice drastically diminishes the response (Marx-Stoelting et al., 2017[48]). Instead, cyproconazole appears to mainly affect human PXR *in vitro* (Luckert et al., 2018[45]). Tumor induction by activators of CAR or the peroxisome proliferator-activated receptor (PPAR) alpha is frequently observed in rodents, while human relevance of the processes occurring downstream these receptors is questioned (Graham and Lake, 2008[21]; Holsapple et al., 2005[29]). Third, toxicokinetic aspects have to be taken into account. *In vivo* dosing can, therefore, not be easily translated into *in vitro* concentrations without having proper *in vivo-in vitro* extrapolation models available for the test compounds. Thus, limited correlation of toxic doses *in vivo* and responses *in vitro* does not necessarily relate to shortcomings of a chosen *in vitro* system and detailed inter-species and toxicokinetic knowledge of individual compounds is necessary to judge on the correlation of *in vitro* data with the outcome of *in vivo* toxicity studies.

However, even if quantitative statements about *in vivo* toxicity remain difficult, the present *in vitro* hepatotoxicity dataset may be helpful in a different context: xenobiotic-induced hepatotoxicity may become manifest in many different ways, such as hepatic cholestasis, steatosis, or hepatocellular necrosis. Future analyses will reveal whether the type of hepatotoxicity, e.g. steatosis, can be predicted using the *in vitro* hepatotoxicity marker panel. Furthermore, the presented approach may be helpful in mixture testing, both with respect to qualitative (e.g. will a certain type of hepatotoxicity be induced by a mixture with sufficient probability but not by the individual compounds alone) and quantitative (e.g. is the mixture more potent than the sum of the individual compounds) aspects. In addition, the parameter of total transcriptional deregulation can be used in mixture testing, for setting up test compound concentrations of a desired potency.

In summary, the present work illustrates the comparability of hepatotoxicity marker testing at the mRNA and protein levels in HepaRG cells. Cellular responses to 30 different pesticides were characterized, providing a basis for future analyses of mechanisms of their toxicity. Furthermore, a number of robust hepatotoxicity marker genes and proteins were identified in HepaRG cells.

## Acknowledgements

The research leading to these results received funding from the German Federal Ministry of Education and Research (BMBF), (e:TOP; Combiomics 2), Grant 031L0118A/ B/C. We would like to thank Julia Sternbeck and Beatrice Rosskopp for their excellent technical support.

## Supplementary Material

Supplementary material

## Figures and Tables

**Table 1:**
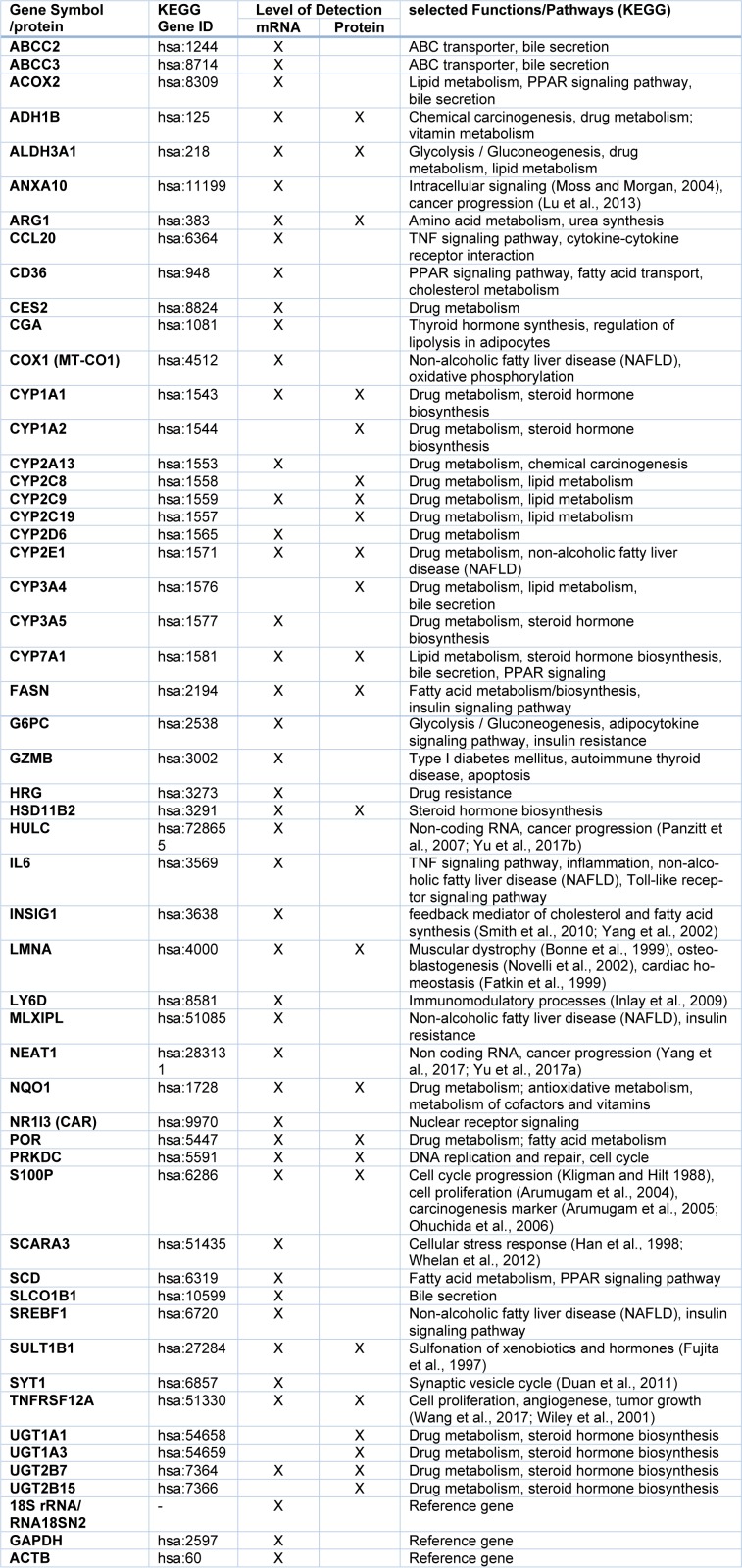
Selection of hepatotoxicity markers and their function

**Table 2:**
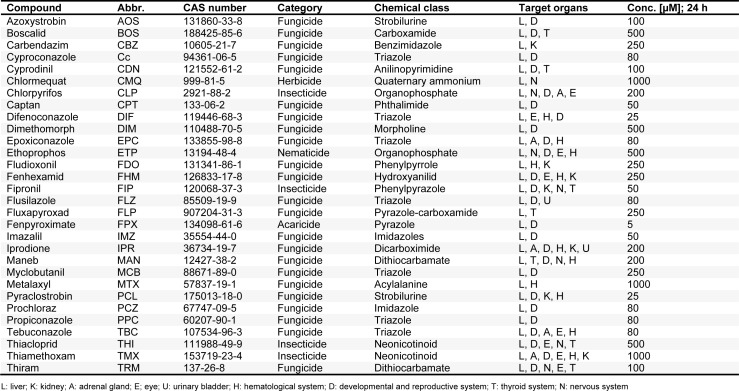
Compound selection and tested concentrations in vitro
